# Psychometric validation of Czech version of the Sport Motivation Scale

**DOI:** 10.1371/journal.pone.0227277

**Published:** 2020-01-02

**Authors:** Martin Komarc, Ivana Harbichová, Lawrence M. Scheier

**Affiliations:** 1 Department of Methodology, Faculty of Physical Education and Sport, Charles University, Prague, Czech Republic; 2 Department of Psychology, Faculty of Physical Education and Sport, Charles University, Prague, Czech Republic; 3 LARS Research Institute, Inc, Scottsdale, Arizona, United States of America; University of Lleida, SPAIN

## Abstract

The Sport Motivation Scale (SMS) is a widely used instrument that assesses motivational processes within sport and exercise environments. The scale has demonstrated validity and reliability in multiple cultures, however, there is yet no empirical evidence regarding its psychometric properties in the Czech population. In this study we therefore set out to examine the reliability and construct validity of the SMS in a sample of Czech university-aged athletes. We first examined the SMS factor structure using a nonparametric item response theory model (Mokken monotone homogeneity model) and identified six items violating the unidimensionality of the particular subscales. Remaining items were then subjected to test of hypothesized seven-factor structure and several different forms of measurement invariance examined based on gender, competition level and type of sport (individual vs team sports). The hypothesized seven-factor fit well and there was sufficient evidence supporting full invariance across the examined groups. All SMS subscales had adequate internal consistencies ranging from 0.66 to 0.89. Results of correlational analysis among the SMS subscales and between the SMS and two outcomes of interest further supported validity of the scale. Observed differences in SMS subscales between males and females, recreational and competitive athletes, as well as between individual and team-based sport activities, comported with prior empirical studies using a self-determination theory framework. In conclusion, results reinforce the utility and performance of the SMS in a sample of Czech university athletes. The SMS may therefore be recommended for measurement of the multidimensional motivational processes taking place in the exercise and sport domain.

## Introduction

The concept of motivation is perhaps one of the most far-reaching constructs in psychology and is fundamental to research on behavior, personality, and individual differences [1]. It is not only scientists that emphasize its importance, even in lay circles, people often mention that one individual is more motivated than another, or they will comment that the winner of a competition seemed more driven. Discussion of motivation can be as simple as “what makes one individual excel at something more than another” or it can be used as an accolade to compliment an individual who works hard at something (e.g., “this person really must like doing this, because they work hard at their craft”). The goal of understanding motivation lies at the core of human values and ripples through how we make sense of the world. Indeed, research has shown beyond a doubt that motivation is crucial to understanding achievement and performance in a variety of settings including education [2], work [3], health and wellness [4] and even parenting [5]. The inability to lose weight, sedentary behavior, and reluctance to engage in behavior change, for instance quitting smoking, can all be ascribed to individual differences in motivation, usually described in terms of a person’s level of commitment, their desire or conviction [6].

The concept of motivation is also fundamental to sport activities, where the results of competition are often ascribed to motivational constructs like “trying harder” or “training with more intensity.” Correct understanding of motivational processes may, for example, spark greater physical activity in those seeking optimal health or improve the training conditions and performance of competitive athletes [7, 8]. Several conceptual frameworks have been designed in order to describe the role of motivation in sport and physical activities. One of these organizing systems, self-determination theory (SDT) [1] appears to be especially useful in understanding an individuals’ degree of involvement in physical activity and is fundamental to sport motivation research [9]. Originally developed as a means of understanding the role of academic motivation in student performance and school`engagement [2], SDT posits that human behavior can be explained on the basis of three psychological needs: competence, relatedness, and autonomy. All three are necessary for optimal functioning and are the basis of ‘eudamonia’ or well-being. Competence deals with curiosity and exploration of the world, seeking challenges and developing confidence. Relatedness is based on belonging, a sense of community and reciprocal care with others. Autonomy addresses a person’s authentic expression, and whether they live in a manner congruent with their values and life goals. The more an individual feels sufficient support for and satisfaction with their competence, relatedness and autonomy, the more they will internalize the standards that produce these behaviors. The end result is they will take responsibility for their actions and feel “motivated” to continue acting in a certain way.

SDT goes one step further to delineate three types of motivation that underlie an individual’s basic needs: intrinsic motivation, extrinsic motivation and amotivation [10]. In the case of sport participation, SDT provides a means to understand individual differences in performance, degree of involvement in physical activity [11, 12], effort and level of commitment. Intrinsic motivation is a tendency to participate in an activity because of the inherent value of the activity itself (e.g., mastery of difficult training techniques). In other words, no extrinsic impetus such as a reward needs to be applied in order to encourage participation, rather it derives from an internal sense of satisfaction. Intrinsic motivation for sport participation is based on inherent interest arising from the pure feeling of enjoyment, pleasure and satisfaction that derive from the physical activity alone and does not require ulterior explanations [13]. Extrinsic motivation refers to behavior controlled by external sources, for instance, when a person engages in sports because it will yield a particular reward (or conversely prevents punishment). Extrinsic motivation is seen as a means to an end and while it may involve some internalization processes, it reflects a diminished sense of autonomy. In most cases, there is less initiative with extrinsic motivation and the individual adheres to or complies with past reward contingencies that are external by nature, ascribing to them an external locus of control. Some individuals are extrinsically motivated to avoid criticism, for instance, an athlete who is pushed during training by a coach who utilizes negative reinforcement to improve performances (i.e., cajoling the athlete to try harder). SDT differentiates four types of extrinsic motivation, based on the degree of relative autonomy of an acting subject: external regulation (the most extrinsically controlled type of motivation), introjection, identification, and integrated regulation (the most autonomous type of extrinsic motivation). In contrast to both intrinsic and extrinsic sources of motivation, amotivation poses there are no linkages between the individual’s actions and outcomes of these actions. In other words, the individual cannot establish reliable contingencies that demonstrate an ability to govern behavior and produce desired outcomes (e.g., I am incapable of succeeding in this sport). Individuals with amotivation don’t value an activity, don’t feel competent to peform it, and based on past experience believe that they cannot control the outcomes.

According to the SDT, amotivation, external regulation, introjection, identification, integrated regulation, and intrinsic motivation represent a self-determination continuum which is bounded by intrinsic motivation on one side and amotivation on the other side [14]. Higher levels on the SD continuum (closer to intrinsic motivation) are often associated with a wide range of positive outcomes including self-efficacy, well-being and positive coping, to name a few [1, 15].

### Assessing motivation in sport from SDT perspective

Studies focusing on applicability of the basic tenets of SDT in exercise and sports have indicated that intrinsic motivation, extrinsic motivation and amotivation are significantly associated with many important antecedents and outcomes related not only to training and performance themselves. In their recent review, Clancy et al. [16] have listed topics and outcomes, which are the most frequently investigated alongside motivation in competitive athletes. Those included: motivational climate (12.7% of included research articles), burnout (12,7%), doping/substance use (9.5%), perfectionism (6.3%), injury (4.8%), and other (e.g. antisocial/prosocial behavior, narcissism, exercise dependence, coping, etc.).

Specifically, research shows that autonomy-supportive coaching may positively contribute to athletes’ motivation, whereas a more controlling style, on the other hand, may undermine motivation, leading to feelings of frustration and anger [17]. Empirical evidence supports a negative association between intrinsic motivation and burnout, and a positive association between amotivation and burnout [18]. Intrinsic motivation can also be protective with regard to risky behaviors, as research shows that high levels of intrinsic motivation in sport is associated with less reported past drug use and future intentions to use drugs [19]. Extrinsically motivated athletes report, for instance, greater alcohol use and more positive doping attitudes than their intrinsically motivated counterparts [20, 21]. Personality also may factor into motivation, with athletes that have higher levels of adaptive perfectionism also reporting more self-determined forms of motivation, while maladaptive perfectionism and non-perfectionism is generally associated with non-autonomous forms of extrinsic motivation and amotivation [22, 23]. Injured intrinsically motivated athletes have exhibited higher engagement in their rehabilitation programs [24]. When they return to competition after an injury, extrinsically motivated athletes experience uneasiness, concerns and worries [25]. To summarize, evidence supports a link between self-determined motivation and adaptive behaviors, whereas maladaptive behavior is linked to extrinsic motivation and amotivation.

This brief overview reinforces the widespread interest in and applicability of motivation research in exercise and sporting contexts. Logically, it follows that a methodologically sound assessment is needed to measure, understand, and predict the influence of motivation in sport and exercise. The SDT has provided a basis to formulate several self-report assessments that attempt to quantify different aspects of motivation (see for example, www.selfdeterminationtheory.org). One of the most frequently used instruments to assess sport and exercise motivation is the Sport Motivation Scale (SMS) [26]. According to Clancy et al. [27], the SMS has the highest citation rate per year (19.5 citations/year) among the most prominent motivation measures in sport, highlighting the importance of this instrument within the sport motivation research community.

### Sport motivation scale

The SMS represents a translation of an earlier French scale– Échelle de Motivation dans les Sports [28]. This was then translated and validated in English [26], resulting in the SMS. The SMS factor structure and scale reliability were orginally determined with a sample of French speaking Canadian university student-athletes, participating in variety of both individual and team sports [26, 28, 29]. The results of both exploratory (EFA) and confirmatory factor analysis (CFA) supported a seven-factor solution. Each latent factor was measured by four non-overlapping items, resulting in a 28-item scale. The seven factors were supposed to represent three types of intrinsic motivation (IM to know, IM to experience stimulation, and IM to accomplish), three types of extrinsic motivation (external regulation, introjection, and identification), and amotivation. Although framed by SDT, the SMS does not include a latent construct assessing integrated regulation, the highest level of internalization of extrinsic motivation [14]. The SMS developers reported adequate reliability (estimated by Cronbach’s alpha) for each factor, as well as moderate to high indices of temporal stability (test-retest reliability).

Since its inception and translation to English, the SMS has undergone considerable scrutiny to ascertain its psychometric properties [26, 28–30]. The bulk of this research has involved university student-athletes although there are a few cases where the instrument has been tested with either professional or competitive athletes or younger age students including children [31–33]. These additional studies following the initial validation provided further evidence of adequate psychometric properties, including a simplex-like pattern between the subscales, construct validity, internal consistency and test-retest reliability [30, 34, 35].

Despite its widespread uses, use of the SMS, however, has not been without criticism. For instance, Martens and Weber [36] reported a relatively poor fit for the seven-factor model that was based on a sample of U.S. college athletes. Several other studies also failed to validate the SMS factor structure using different age groups and non-English native speaker athletes [37–39]. In addition, several studies suggest that the internal consistency of the SMS subscales is less than optimal [40]. Additional criticisms have highlighted that the SMS does not include a measure of integrated regulation, making it impossible to tap all of theoretical constructs proposed by SDT. Several studies have focused on the utility of using a three-factor conceptualization of IM within SMS. Some authors [36, 40] have reported problems with discriminant validity of the IM subscales, given their relatively high magnitude of association. Others [41] have reported that using a three-factor IM solution lowered validity coefficients with motivational consequences, compared to studies using a general IM scale. Moreover, the departure of SMS from the SDT framework has created opportunities to develop an assessment instrument containing all forms of extrinsic motivation (including integrated regulation) and only one general IM scale [30, 40–42].

Notwithstanding these concerns, the SMS has been widely used over the last few years and fostered a considerable emphasis on understanding motivational processes taking place in exercise and sport context [30]. The instrument’s influence has been greatly enhanced by several foreign language translations, including Bulgarian, Spanish, Portuguese, German, Italian, Greek, Serbian, Russian, Turkish, Arabic and Hungarian [32, 43–51]. The availability of the SMS in different languages provides a means to assess sport motivation and the relevance of SDT across different cultures. Notwithstanding, a rigorous test of psychometric properties of the SMS in Czech population, where competitive sports are highly valued and well supported, is still absent.

### Exploring group differences in sport motivation

Researchers have also shown tremendous interest in examining group differences in the motivational constructs assessed by the SMS. This has included whether there are gender differences in sport motivation. For instance [26, 28, 29] reported that female athletes scored higher on intrinsic motivation and lower on extrinsic motivation and amotivation. Separately, [44, 52, 53] reported that female athletes scored lower on extrinsic motivation and amotivation than males, but did not differ significantly on intrinsic motivation. Interestingly, De Pero et al. [15] found no evidence of gender differences in any of the SMS subscales using a sample of older Italian athletes. Studies conducted with young Malaysian athletes indicated males had higher levels of IM as well as higher levels of extrinsic motivation than females [54, 55]. The same results were observed by Filho et al. [46], who validated the SMS using a sample of university-aged athletes in Brazil. Notwithstanding these findings, some authors have concluded that compared to their male counterparts, female athletes participate in sport and exercise activities because of enjoyment, satisfaction and pleasure, rather than because of extrinsic reasons.

Several studies have assessed the effect of competition level on motivation in sport [29, 32, 37, 45]. According to SDT, high pressures (winning, or outperforming others) and rewards (e.g. financial) in competitive sport may alter an athlete’s perceived locus of control (from internal to external) and in turn diminish IM and lower their self-determination [56]. A number of studies have supported this premise with young athletes, while young recreational athletes [29] and non-scholarship athletes [57, 58] exhibited higher levels of IM than their same-age competitive and scholarship counterparts, respectively. However, Brodkin and Weiss [59] reported that the influence of competition level on IM in sport is much more salient in younger athletes than in older adults. A study by DePero et al. [37] revealed that senior elite competitors (over 65 years) are more intrinsically motivated than senior non-elite competitors, highlighting specific age-related perceptions of rewards and pressures present in competitive sport. Similarly, somewhat undermining SDT, Teo and colleagues [55] reported that IM did not significantly differ between young competitive and casual bowlers from Malaysia.

A third relevant area of inquiry considers the role of individual versus team-based sport activities and their relative effect on psychological functioning [60]. SDT posits that motivation for an activity depends on the extent to which the activity satisfies three basic psychological needs (autonomy, satisfaction, and relatedness). It is clear that motivation for individual and team sports may thus vary greatly in satisfying these needs. Some authors have suggested that team-based sports provide greater psychosocial benefits, compared to individual sports, probably because individuals participating in team sports are more exposed to prosocial peers and favorable or esteem-enhancing social interactions [61], providing greater opportunities to satisfy the need of relatedness. Some support for this contention was provided by Nielsen et al. [62], who conducted focus group interviews with middle-aged athletes and found that participation in team sports like football is associated with higher levels of intrinsic motivation, compared to engaging in individual fitness activities and spinning. Recently, Wikman, et al. [63] observed similar results, while assessing the effect of floorball and spinning classes/training on motivation using the SMS in a sample of middle-aged women. Although the differences in motivation underlying individual and team sports might be of great importance in verifying the basic tenets of SDT, studies assessing these differences with the SMS are rarely conducted [30].

### Focus of the present study

Despite the demonstrated utility of SDT in understanding motivation for engaging in sports and exercise, there is yet no valid instrument able to assess the theoretical constructs proposed by this theory in the Czech culture. The Czech culture is greatly enamored with sports and exercise, with professional team competing at high levels in many different sports (hockey and soccer, to name a few) and a strong focus on youth sport development. This makes it important for Czech researchers, coaches, and sport psychologists to have a reliable means to assess key motivational constructs related to sport and exercise. To address this concern, we examine the psychometric properties of the SMS in a sample of physically active Czech university students. First, we examined the dimensionality of a Czech translated version of the SMS using nonparametric item response theory (IRT) models and then tested the instrument’s factorial validity using the confirmatory factor analysis (CFA) approach. Following accepted conventions for testing measurement invariance (e.g., [64]), we then examined model equivalence across subgroups of gender, sports (individual vs. team), and also competition levels (highly competitive, recreational). All three of these sample characteristics have been tied to observed subgroup differences in sport motivation and it should be emphasized, that a certain form of measurement invariance (e.g., scalar invariance) constitutes an important methodological prerequisite of a correct interpretation of such differences [65]. This prerequisite, however, was almost never empirically verified in the SMS validation studies. Finally, we examined the relations of the SMS subscales with outcome measures, as well as we tested group differences in motivation, again based on gender, type of sport and competition level. Collectively, the findings of this study should yield preliminary information on the suitability of the SMS for the Czech culture as well as provide an important basis for future cross-cultural comparisons of motivation in sport.

## Methods

### Participants

A sample of 456 undergraduate students (290 males, 166 females) with mean age of 21.6 years (SD = 2.1) was drawn from the Faculty of Physical Education and Sport, Charles University in Prague, Czech Republic. Participants’ experience with the most current sport activity ranged from 1 to 21 years (M = 10.2, SD = 4.7) and the most of the sample (55.9%) trained at least four times per week. The sample contained 51.4% individuals participating in a variety of individual sports (e.g., running, swimming, skiing, tennis, etc.) and 46.6% participating in team-based sports (e.g. football, basketball, florbal, ice-hockey etc.). Participants were classified according to their competition level as either highly competitive (31.6%) or recreational (68.4%) athletes. The group of highly competitive athletes constituted athletes competing at the highest national (e.g., national championship, the highest national league), or international level (e.g. Olympic games, World championship, Champion’s league), whereas the remaining participants were grouped as recreational athletes.

Recruitment strategies involved convenient sampling methods taking place during sport psychology seminars at the Department of Psychology, Charles University, located in Prague. During the consenting procedure, all participants were informed about the purpose of the study and of their ethical rights. The study was voluntary and anonymous and participants were free to terminate their participation in the study at any time resulting in no penalty. By returning the questionnaire to the first author, participants consented with the processing of their data for research purposes. Ethics Committee at the Faculty of Physical Education and Sport, Charles University in Prague did not provide formal approval for studies using exclusively anonymous questionnaires and inventories at the time of data collection.

### Measures

#### Sport motivation scale (SMS)

In this study the Czech version of the SMS was administered. The Czech version of the SMS was developed using a modified direct translation procedure in combination with protocol analysis [66]. Three independent bilingual individuals (with backgrounds in general and/or sport psychology) translated the original SMS from the English to Czech language. Instructions for the translators emphasized that the literal translation was not necessary, rather they should capture the Czech meaning of the original SMS items. The three translated versions were next discussed at a meeting of all translators along with the first author (IH). The discussion focused on lexical differences of words, idiomatic expressions and specific sport terminology between the English and Czech languages. A consensus was reached regarding the best translated version for each item producing the initial Czech language version of the SMS. To ensure the semantic equivalence of the translated version we next used a protocol analysis [67]. The initial Czech version of the SMS was administered to 10 physically active university students (5 males, 5 females), who were then interviewed to ensure that they understood both the instructions to complete the instrument, and the wording of each SMS item. The students were also asked to explain their responses and understanding of the SMS items. This qualitative information was used to create the final Czech version of the SMS, which was utilized in the present study examining the psychometric properties of the scale–see S1 Appendix. The translated Czech version of the SMS has been shown to have sufficient semantic equivalency with respect to the English version [68].

The SMS contains seven subscales, which are supposed to measure three types of intrinsic motivation (to know, to experience stimulation, to accomplish), three types of extrinsic motivation (external regulation, introjection, identification), and amotivation. There are four items per subscale resulting in a total of 28 items in the scale. Respondents were asked about the reasons for their participation in sports and their responses were quantified on a 7-point Likert response scale ranging from 1 (‘does not correspond at all’) to 7 (‘corresponds exactly’). Scales were scored toward higher levels of a particular type of motivation.

#### Outcome measures

We examined convergent validity of the SMS sub-scales using two other theoretically relevant constructs–physical self-worth (PSW), and global self-esteem (GSE)–both major factors related to motivation in sport and exercise domain [69–71].

PSW was measured by six-item subscale from the Physical Self-Perception Profile (PSPP) [72]. The questionnaire employs a forced-choice alternative format to avoid socially desired responses. For each item there are two statements (e.g., some people are very competitive vs. others are not quite so competitive), each with two possible alternatives (‘sort of true,’ and ‘really true’). We assessed GSE using the Rosenberg Self-Esteem Scale (RSES) [73]. The RSES contains 10 items, five of which are positively framed (e.g., ‘On the whole, I am satisfied with myself’) and five that are negatively framed (e.g., ‘At times I think I am no good at all’). The scale uses a four-point Likert scale response format (1 = ‘strongly agree,’ 4 = ‘strongly disagree’). Both instruments have been applied in the Czech population [74–76], are frequently used in studies of sports participation and exercise, and have excellent psychometric properties [77–79]. Theoretical tenets of the SDT suggest, that autonomous forms of motivation (e.g. intrinsic motivation) should be positively related to both outcome measures, whereas a negative association is expected between amotivation and the outcomes.

### Analysis strategy

We first examined the dimensionality of each SMS subscale using nonparametric IRT approach. Specifically, we used the Mokken’s monotone homogeneity (MH) model, which is intended for scaling items on a unidimensional, ordinal scale [80]. Although the popularity of Mokken scale analysis has rapidly grown over the past decade among researchers from many different research areas [81], this approach has not yet been applied in SMS validation stdies. The major advantage of the Mokken’s MH model lies in its absence of parametric assumptions for describing associations between individual item responses and underlying latent variable (e.g. S-shaped curve traditionally used with parametric IRT models such as 1-parapeter logistic model–[82]). Mokken’s MH model is considered appropriate to conduct a unidimensionality check in situations when a large number of different constructs are measured by a small number of indicators [81]. A basic premise in testing and constructing a Mokken scale is the role of homogeneity coefficients (also referred to as scalability coefficients, [81, 83]), which, for a particular item *i*, are denoted as *H*_*i*_, and are defined as the ratio of the sum of all item’s observed covariances over the sum of all item’s maximal covariances. We calculated *H*_*i*_, for each item within all seven SMS subscales using the package “mokken” available in the freeware statistical computing environment R [84]. As a rule of thumb, Mokken [85] recommends that in unidimensional scales each item’s *H*_*i*_ > 0.3, but a cut-off *H*_*i*_ > 0.4 may be used in order to obtain higher certainty of the MH model fit. Thus the value of *H*_*i*_ > 0.4 was chosen as a cut-off for subscales of intrinsic motivation, given the proportionally larger number of items measuring intrinsic motivation in the SMS. In all other SMS subscales we considered items with *H*_*i*_ < 0.3 as those violating the assumption of unidimensionality. Items with low scalability coefficient were excluded from subsequent test of the hypothesized latent structure of the SMS.

We then tested the hypothesized seven factor structure of the SMS using CFA with the Mplus program version 7.4 [86]. As a methodological refinement, we analyzed the SMS items as ordered categorical variables, the approach which is usually omitted in most of the validation studies of the SMS. Traditionally, SMS items, although categorical in nature (7-point Likert scale), have been analyzed as interval continuous variables. Pearson product-moment correlations between all pairs of SMS items have then been considered as a sufficient statistics for factor analysis (e.g., CFA) if the items follow a multivariate normal distribution (a requirement, which is very difficult to fulfill in practical situations and was almost never tested in SMS validation studies). However, methodological research has indicated that using this approach with ordinal data can results in biased parameter estimates (e.g. factor loadings) as well as in problems with parameters interpretation [87]. In a categorical CFA model we assume that ordered-categorical item responses are discrete representations of continuous latent variables. The proportion of individuals who endorse each categorical response option provides information about the latent response distribution by way of threshold (τ) parameters. Therefore, unlike in classical CFA where we estimate one intercept per item, we estimate *k*-1 (where *k* is the number of response categories) thresholds for each item in categorical CFA. As recommended, we used the WLSMV estimator with robust standard errors, an estimator which does not assume normally distributed variables and provides the best option for modeling ordered categorical data [88]. The WLSMV estimator estimates models with missing data (there were less than 1% of missing data–see S1 Dataset) based on the full sample (N = 456) and the full information that is available using pairwise present methods [89]. Latent factor scores were estimated using the maximum of the posterior distribution of the factor, which is also referred to as the Maximum A Posteriori (MAP) method [90]. The CFA model fit was evaluated using the χ^2^ test statistic, and given its sensitivity to sample size, we also used other inferential fit indices including the root mean square error of approximation (RMSEA) [91], Comparative Fit Index (CFI) [92], and Tucker-Lewis Index (TLI) [93]. Both the TLI and CFI have benchmarks close to 1.0 (all of the sample variance and covariances are accounted for by the implied population model) with acceptable fit indicated by values > 0.95 [94]. The ratio of the model chi-square to its degrees of freedom should be less than 3 and the RMSEA should approximate 0.06 or less in good-fitting models [95].

We next used multi-group CFA to examine measurement invariance across gender, competition level, and type of sport. As a first step, we assessed configural invariance determining whether the hypothesized latent factor structure fits the observed data in each group (the same number of factors exists in each group with no further constraints). We then progressively imposed additional parameter restrictions including metric (factor loading are set equal in compared groups), scalar (items thresholds are set equal), and finally full invariance setting also the residual terms to equality. The nested comparisons using a χ^2^ difference test always involved the more restrictive model containing the parameter constraints compared to the less restrictive model with freely estimated parameters [95].

Subsequently, Pearson’s correlation coefficient was used to express the relationship between SMS subscales and factor scores and outcome measures. Group differences in SMS subscales were assessed using independent group t-test. Reliability of the SMS subscale factor scores was estimated by McDonald’s ω, an estimator based on the common factor model [96]. Based on Monte Carlo simulation efforts [97] we have sufficient number of observations to achieve precise parameter estimates with adequate power > .80. This determination was based on using 10,000 replications with ML estimation and using the criteria of adequate coverage (the 95% Confidence Interval contains the parameter), low standard error bias, and a high proportion of replications where we can reject the null specifying the parameter is zero at the 0.05 alpha level.

## Results

### Mokken scale analysis of the SMS items

Table 1 contains scalability coefficients along with the means and standard deviations for the SMS items presented within each subscale. Three out of nine items measuring different types of intrinsic motivation (items 2, 4 and 1) did not reach the required cut-off value for the *H*_*i*_, coefficient. Unacceptable values of scalability coefficients (*H*_*i*_ < 0.3) were further observed in one item from each of the following SMS subscales: identification (item 24), introjection (SMS9), and amotivation (SMS19). These six items violating the unidimensionality requirement were excluded from the subsequent CFA, since subscale unidimensionality is consistent with the logical requirement of simple structure for a well-fitting CFA model.

**Table 1:** SMS item means (standard deviations), scalability coefficients (*H*_*i*_) and standardized factor loadings from CFA model.

Item	Mean (SD)	*H*_*i*_	IM-K	IM-A	IM-E	IDE	INT	ETR	AMO
Item 2^#^	4.19 (1.62)	0.32 (0.03)							
Item 4^#^	4.26 (1.59)	0.39 (0.03)							
Item 23	4.90 (1.50)	0.44 (0.03)	0.79						
Item 27	4.17 (1.69)	0.40 (0.03)	0.66						
Item 8	5.42 (1.38)	0.41 (0.03)		0.70					
Item 12	5.46 (1.24)	0.44 (0.03)		0.76					
Item 15	5.90 (1.11)	0.46 (0.03)		0.79					
Item 20	4.94 (1.50)	0.47 (0.03)		0.83					
Item 1^#^	5.69 (1.18)	0.38 (0.03)							
Item 13	5.81 (1.23)	0.42 (0.03)			0.86				
Item 18	5.72 (1.23)	0.44 (0.03)			0.85				
Item 25	5.59 (1.28)	0.42 (0.03)			0.85				
Item 7	4.34 (1.64)	0.36 (0.03)				0.43			
Item 11	5.23 (1.37)	0.31 (0.04)				0.77			
Item 17	4.72 (1.46)	0.31 (0.04)				0.65			
Item 24^#^	3.87 (1.71)	0.29 (0.04)							
Item 9^#^	5.27 (1.46)	0.28 (0.04)							
Item 14	5.24 (1.52)	0.46 (0.03)					0.85		
Item 21	5.27 (1.67)	0.40 (0.03)					0.66		
Item 26	4.27 (2.13)	0.35 (0.03)					0.60		
Item 6	3.18 (1.66)	0.44 (0.03)						0.63	
Item 10	3.70 (1.69)	0.47 (0.03)						0.77	
Item 16	2.59 (1.47)	0.40 (0.04)						0.60	
Item 22	3.69 (1.69)	0.48 (0.03)						0.71	
Item 3	2.10 (1.52)	0.40 (0.04)							0.76
Item 5	1.95 (1.31)	0.45 (0.04)							0.77
Item 19^#^	1.23 (0.75)	0.27 (0.07)							
Item 28	2.25 (1.37)	0.45 (0.04)							0.72

Note: IM-K = intrinsic motivation to know, IM-A = intrinsic motivation to accomplish, IM-E = intrinsic motivation to experience stimulation, IDE = identified regulation, INT = introjection, EXT = external regulation, AMO = amotivation,

^#^ -item not included in CFA due to low H coefficient

### Results of CFA

Following tests of the Mokken MH model, we then tested the seven-factor CFA model. This model fit the observed data well, χ^2^(188) = 478.5, p < 0.001, χ^2^/df = 2.5, RMSEA = 0.059, TLI = 0.95, CFI = 0.96. The standardized factor loadings (listed in Table 1) varied from a low of .43 to a high of .86 (average λ = .73). The strongest inter-factor correlations (Table 2) were observed between scales measuring IM (r = .0.62 to 0.86) suggesting these scales might tap very similar underlying latent constructs. Nevertheless, the overall pattern of factor correlations provided support for a simplex ordering of the SMS subscales based on the larger magnitude of relations for within-construct versus between-construct subscales. To illustrate, amotivation had a significant positive association with external regulation, did not correlate significantly with introjection, and correlated negatively with identification and intrinsic motivation subscales. The estimates of internal consistency based on the common factor model (McDonald ‘s ω) for each SMS subscale ranged from a low of 0.66 to a high of 0.89. Overall, the CFA model results support the hypothesized latent factor structure and reinforced the construct validity of the SMS in our sample.

**Table 2:** Inter-factors correlations (lower diagonal), McDonald’s ω (diagonal), means and standard deviations for hypothesized seven-factor model of SMS.

	1	2	3	4	5	6	7	M (SD)
1. IM-K	*0*.*69*							4.54 (1.38)
2. IM-A	0.86	*0*.*85*						5.43 (1.05)
3. IM-E	0.62	0.75	*0*.*89*					5.70 (1.09)
4. IDE	0.60	0.63	0.50	*0*.*66*				4.76 (1.11)
5. INT	0.36	0.50	0.39	0.34	*0*.*75*			4.93 (1.40)
6. EXT	0.35	0.23	0.18	0.37	0.32	*0*.*78*		3.29 (1.23)
7. AMO	-0.13	-0.30	-0.31	-0.14	-0.05	0.38	*0*.*80*	2.10 (1.11)

Note: IM-K = intrinsic motivation to know, IM-A = intrinsic motivation to accomplish, IM-E = intrinsic motivation to experience stimulation, IDE = identified regulation, INT = introjection, EXT = external regulation, AMO = amotivation

### Invariance tests

We next conducted invariance tests (configural, metric, scalar, strict/full) of the hypothesized SMS model across gender, competition level, and type of sport. Table 3 contains the fit indices for the full set of invariance models.

**Table 3:** Fit indices for measurement invariance tests.

	χ^2^	d.f.	p	RMSEA	CFI	TLI	Δ χ^2^	Δ d.f.	Δ p
**Full sample**	478.5	188	0.000	0.058	0.96	0.95			
**Gender (male, female)**									
Configural invariance	677.4	376	0.000	0.060	0.96	0.95			
Metric invariance	713.7	406	0.000	0.058	0.96	0.95	35.8	30	0.215
Scalar invariance	832.1	486	0.000	0.056	0.95	0.95	118.4	80	0.003
Full invariance	837.2	508	0.000	0.053	0.95	0.96	5.1	22	1.000
**Sport (individual, team)**									
Configural invariance	660.8	376	0.000	0.058	0.96	0.95			
Metric invariance	689.2	406	0.000	0.056	0.96	0.95	28.4	30	0.549
Scalar invariance	782.0	486	0.000	0.052	0.96	0.96	92.8	80	0.155
Full invariance	805.7	508	0.000	0.051	0.96	0.96	23.7	22	0.363
**Level (recreational, competitive)**									
Configural invariance	704.8	376	0.000	0.062	0.95	0.94			
Metric invariance	729.2	406	0.000	0.059	0.95	0.95	24.4	30	0.754
Scalar invariance	818.3	486	0.000	0.055	0.95	0.95	89.1	80	0.228
Full invariance	844.7	508	0.000	0.054	0.95	0.95	26.4	22	0.235

Note:d.f. = degrees of freedom; RMSEA = Root Mean Square Error of Approximation; CFI = Comparative Fit Index; TLI = Tucker Lewis Index; Δ = change with respect to less restricted model.

For all three grouping variables, the baseline configural model positing the same configuration of factors across the groups exhibited good fit with the data. The introduction of equality constraints on factor loadings (i.e., *λ*_*i*_) indicated no significant deterioration of the model fit (gender: Δχ^2^_30_ = 28.5, p = 0.544; sport: Δχ^2^_30_ = 28.4, p = 0.549; competition level: Δχ^2^_30_ = 24.4, p = 0.754), suggesting that the factor loading are equivalent across subgroups. Further restrictions of item thresholds (τ_i_) resulted in scalar invariance models that, within each of the grouping variable, exhibited excellent model fit according to the both incremental (CFI, TLI) and absolute (RMSEA, χ^2^/df) fit indices. For the gender analysis, the formal nested Δχ^2^ test between scalar and metric invariance models was statistically significant, Δχ^2^(80) = 118.4, p = 0.003. Other comparative fit indices (e.g., ΔCFI), however, fell within the various benchmarks supporting invariance (the difference should be less than .01, e.g., [98], indicating that the scalar invariance holds for all three grouping variables, including gender. Nonsignificant differences between full invariance models with less restricted scalar invariance models revealed that equal item residuals (i.e. *θ*_*i*_) across groups is also a plausible hypothesis within each grouping variable (gender: Δχ^2^_22_ = 5.1, p = 1.000; sport: Δχ^2^_22_ = 23.7, p = 0.363; competition level: Δχ^2^_22_ = 26.4, p = 0.235). Taken together these results support the full measurement invariance between males and females, individual and team sports as well as between recreational and competitive athletes–an important requirement of valid between group comparisons using the observed SMS subscale scores.

### Association with outcome measures

We next assessed the construct validity of the SMS subscale scores with two other theoretically relevant measures including physical self-worth (PSW), and general self-esteem (GSE). Table 4 contains the results of these bivariate associations using the observed scale composite scores (left part) and the CFA latent construct scores (right part). According to SDT, both the GSE and PSW should be positively associated with autonomous forms of motivations and negatively associated with amotivation. As depicted, GSE was significantly associated with IM in the hypothesized direction with correlations ranging from r = 0.12 to 0.20. Similar results, although larger in magnitude, were observed for PSW (r = 0.18 to 0.27). Extrinsic motivation subscales were significantly related to PSW, but not to GSE. As expected, amotivation was negatively correlated with both outcomes.

**Table 4:** Correlations of SMS subscales with outcome measures.

	Scale scores		Factor scores	
	GSE	PSW	GSE	PSW
**Intrinsic motivation**				
To know	0.12*	0.19**	0.12*	0.18**
To accomplish	0.15**	0.22**	0.16**	0.27**
To experience stimulation	0.17**	0.22**	0.20**	0.26**
Intrinsic combined	0.15**	0.22**	0.18**	0.27**
**Extrinsic motivation**				
Identification	0.08	0.16**	0.06	0.13*
Introjection	0.03	0.13**	0.02	0.12*
External regulation	-0.05	0.13**	-0.05	0.18**
Extrinsic combined	0.02	0.17**	0.01	0.19**
**Amotivation**				
Amotivation	-0.30**	-0.18**	-0.39**	-0.27**

Note:

* p < 0.05;

** p < 0.01,

GSE = global self-esteem, PSW = physical self-worth.

### Group differences in SMS subscales

We next tested subgroup differences in sport motivation using the observed (average) SMS subscale scores. Table 5 contains the results of these analyses. Female participants reported significantly higher levels of identified regulation (p = 0.008), the most autonomous type of extrinsic motivation measured by the SMS. Male athletes, on the other hand, reported significantly higher mean scores in external regulation (p = 0.004) and amotivation (p = 0.010). Comparison involving type of sport revealed that athletes participating in individual sport activities reported higher levels of identified regulation (p = 0.016), whereas team-based athletes reported significantly higher scores in external regulation (p = 0.014). Recreational athletes did not significantly differ from competitive athletes on any of the SMS subscale.

**Table 5:** Mean (SD) differences in SMS subscales by gender, competition level and type of sport.

	Gender			Competition level			Type of sport		
	Male	Female	p	Recreational	Competitive	p	Individual	Team	p
1. IM-K	4.54 (1.32)	4.53 (1.47)	0.925	4.52 (1.34)	4.56 (1.46)	0.801	4.53 (1.39)	4.54 (1.37)	0.933
2. IM-A	5.44 (1.01)	5.42 (1.12)	0.851	5.41 (1.07)	5.48 (1.02)	0.492	5.52 (1.00)	5.34 (1.11)	0.072
3. IM-E	5.70 (1.07)	5.72 (1.13)	0.848	5.72 (1.08)	5.68 (1.11)	0.709	5.69 (1.08)	5.72 (1.10)	0.770
4. IDE	**4.66 (1.12)**	**4.95 (1.07)**	**0.008**	4.72 (1.09)	4.86 (1.15)	0.208	**4.89 (1.05)**	**4.64 (1.16)**	**0.016**
5. INT	4.90 (1.39)	4.97 (1.40)	0.653	4.97 (1.34)	4.84 (1.51)	0.369	4.80 (1.42)	5.06 (1.36)	0.051
6. EXT	**3.41 (1.18)**	**3.07 (1.29)**	**0.004**	3.24 (1.16)	3.41 (1.38)	0.171	**3.15 (1.27)**	**3.44 (1.18)**	**0.014**
7. AMO	**2.20 (1.15)**	**1.92 (1.02)**	**0.010**	2.10 (1.12)	2.08 (1.11)	0.837	2.03 (1.09)	2.17 (1.13)	0.173

Note: IM-K = intrinsic motivation to know, IM-A = intrinsic motivation to accomplish, IM-E = intrinsic motivation to experience stimulation, IDE = identified regulation, INT = introjection, EXT = external regulation, AMO = amotivation

## Discussion

This study provides initial evidence of the utility of a Czech translated version of the SMS to assess sports motivation according to the tenets of SDT. The nonparametric IRT tests identified six items violating the required unidimensionality criteria for a subscale. This is consistent with other studies that have identified problematic items that surface when adapting the English version of the SMS to non-English cultures [37, 44, 46]. The Mokken scale analysis indicated that items 1, 2, 4, 9, 19 and 24 lack validity with respect to the proposed underlying factors with Czech university students and were excluded from further analysis on this basis.

Results of the CFA with the remaining 22 SMS items supported the hypothesized seven-factor structure. The item to factor loadings were all significant and moderate in size showing that we hypothesized the model correctly for this sample of Czech students. The good fit of the model may be partly attributable to trimming the six problematic items from the scale, based on the previous results of the Mokken scale analysis. These results are in line with a number of studies indicating, that the model fit is usually distorted by few individual items and that the core of the SMS exhibits a clear and expected latent structure [37, 44, 46].

Reliability estimates for the SMS subscales were also in the acceptable range (mean ω = 0.77) and comparable to those reported in original validation studies with US and Canadian university students [26, 28, 29]. In our case we observed two SMS subscales with ω < 0.7, namely IM to know (ω = 0.69), and introjected regulation (ω = 0.66). It has to be noted, however, that the IM to know scale only had two items and the introjected regulation scale had three items, perhaps curtailing the estimates with so few items. Reliability estimates using the full set of four items, as originally intended for these scales, would improve to .82 and .71 for IM to know and introjected regulation, respectively (these estimates were derived using the Spearman–Brown prophecy formula, which describes the relationship between test length and reliability and has the following form: $\rho^{*}\frac{n\rho^{'}}{1+(n-1)\rho^{'}}$, where ρ* is the predicted reliability for the new test, *n* is the length of the new test (e.g. *n* = 2 means doubling of the actual test length), and *ρ*′ is the reliability of the current test [96]). Even with these two problematic scales, overall, we can conclude that the SMS subscales have adequate internal consistency.

The pattern of associations between SMS subconstructs matched closely findings from other studies [36, 37, 53]. The inter-factor correlations support the desired simplex-like pattern between the SMS subscales. Overall, most of the associations were in expected directions (e.g. negative association between amotivation and intrinsic motivation) and reached expected magnitudes with the exception of identified regulation, which had a trivially larger magnitude of association with external regulation (r = 0.37) than with introjection (r = 0.34). The evidence would seem to indicate that the SMS subscales are arranged on a continuum in keeping with SDT. Our results are also in keeping with previous empirical findings regarding the discriminant validity of the SMS IM subscales. Indeed, there has been a considerable discussion regarding the utility of using a three-factor conceptualization of IM, particularly given the large amount of shared variance in the three IM subscales [30, 35, 36, 40, 41]. This has led to creation of instrument assessing sport motivation containing only one general IM scale [40–42]. Nevertheless, the SMS with three distinct IM subscales might be especially useful for examining the different forms of IM and their influence on behavior in exercise and sport contexts [30].

We also obtained consistent evidence of measurement invariance across gender, competition level, and type of sport. This provides a sound psychometric basis for valid and meaningful comparisons of the latent scores between the subgroups we examined [65]. Although other studies have examined gender invariance, we believe that we provide the first evidence of invariance contrasting individual and team sport activities. The fact, that the SMS subscales are relevant for both the individual and team-based sports, may be especially important in light of the recent increased interest in differences between these two types of sport activities and their role in behavior regulation [61, 63].

By all accounts the pattern of associations between SMS subscales and outcome measures indicated that the scale preforms as expected according to SDT. Past findings have shown that intrinsic motivation is associated with positive consequences, and amotivation is associated with negative outcomes [16]. We confirmed this expectation given the significant positive associations between IM subscales and high self-related evaluations at both the contextual (physical self-worth) and global (global self-esteem) level. The negative associations were observed between amotivation and the outcomes. The observed pattern of correlations between the SMS and outcome measures also gives support to Hagger & Chatzisarantis’s [7] idea, that context-specific motivation (e.g. sport motivation) should be more strongly related with the context-specific consequences (e.g. physical self-worth) than with the consequences situated at a global level (e.g. global self-esteem). The current findings suggest there is sufficient convergent validity of the SMS test scores when used with Czech students.

The comparison of mean levels of the motivational subscales indicated some gender dissimilarity with female athletes scoring higher than male athletes on the identified regulation subscale, but lower on the external regulation and amotivation subscales. This finding is in line with the majority of studies examining gender differences in sport motivation [26, 28, 45] and is consistent with the suggestion that extrinsic motives for sport participation is more salient for male athletes.

Lack of significant differences in sport motivation between recreational and highly competitive athletes in our study might seem contradictory with respect to previous research, which suggests that pressures and rewards related to participating in a competitive environment may negatively influence IM [29]. Similar to our results and somewhat undermining SDT, Teo and colleagues [30] recently reported that IM did not significantly differ between a cohort of Malaysian young competitive and casual bowlers. This inconsistency suggests that additional research is needed to clarify whether there are stable differences in motivation with respect to athletes’ competition level and also elucidate factors that may contribute to these differences. It should be noted, however, that differences in the categorization schemes used to classify athletes as competitive versus recreational/casual/ non-elite athletes may contribute to the different empirical findings. Moreover, we believe that rewards and pressures can simply be interpreted and perceived by athletes as having more informational than a controlling role even in a competitive environment, leaving their motivation undistorted [56, 57].

Our investigation of motivation levels associated with the participation in team versus individual sport activities revealed that participants from individual sports scored higher in identified regulation, while athletes participating in team sports exhibited higher external regulation. This finding fits with SDT, suggesting that athletes who participate in sports because they feel their involvement contributes to their growth and development as a person represent an example of identified motivation [26]. This is very likely to be the case in individual sport activities, as individual sport involvement is predominantly associated with better physical functioning and less with social adjustment, when compared to team-based sport involvement [61]. External regulation on the other hand, captures behavior controlled by external sources, such as constraints imposed by others [69]. In keeping with the SDT conceptual framework we suggest that the social aspects of sport participation, including such factors as increased interactivity [99], gaining new friends [63], team belonging [62], and pursuit of a common goal [100], may be the main reasons for increased external regulation in team-based sport athletes. However, our findings, including the nonsignificant differences in IM, lend little support to Eime’s et al. [61] supposition, that participating in team-based sports holds a clear advantage for positive psychological outcomes over individual sports. When discussing the differences in motivation and its antecedents between individual and team sports, we shall keep in mind, that training and workouts in many individual sports takes place in group settings. Satisfaction of the basic psychological needs (autonomy, competence, and especially relatedness) in individual sports may therefore be comparable to team sports, pointing to the importance of controlling for the need satisfaction in order to obtain valid comparisons between these types of sports.

There are several limitations in the present study that should be noted. We did not address some of the important psychometric properties of the Czech SMS, including cross-validation or test-retest reliability. Test-retest requires additional data collection involving a repeated measures design and cross-validation requires a larger sample for appropriate power. The cross-sectional design does not allow causal inferences regarding the effects of SMS on outcomes nor can we assess individual change in motivation as well as factors that contribute to change over time. Future studies may want to rely on longitudinal designs in order to examine developmental trends in motivation and include a wider array of outcomes such as sport engagement, school drop-out, academic performance, and related performance measures. Finally we conducted this study with university students quite homogenous in age and who are ideally situated for participation in sports and physical activities. Future studies may want to validate the psychometric properties of the Czech SMS using either younger or older athletes, as no valid instrument is currently available for measurement of sport motivation in both of these age groups.

In conclusion, the findings of the present study provided support for the latent structure, factor validity and reliability of the Czech version of the SMS when applied to university athletes. The findings have ramifications for both the theoretical and practical issues related to scale development. First, our findings contribute to sport motivation measurement theory by being the first to establish the measurement invariance of the SMS across individual and team sports. Intervention programs intending to boost motivation can utilize this information as they target important subgroups. From a practical perspective, Czech researchers, sports psychologists and coaches can now be more confident in using the SMS to measure and better understand the multidimensional motivational processes taking place in exercise and sport domain.

## Supporting information

S1 Appendix English and Czech versions of the SMS. (XLSX)

S1 Dataset (XLSX)
